# A combination technique of strip free gingival grafts and xenogeneic collagen matrix in augmenting keratinized mucosa around dental implants: a single-arm clinical trial

**DOI:** 10.1186/s12903-024-04184-y

**Published:** 2024-05-29

**Authors:** Jia-Ping Huang, Yi-Yu Wang, Anna Dai, Ping Sun, Pei-Hui Ding

**Affiliations:** 1 Stomatology Hospital, School of Stomatology, Zhejiang University School of Medicine, Zhejiang Provincial Clinical Research Center for Oral Diseases, Key Laboratory of Oral Biomedical Research of Zhejiang Province, Cancer Center of Zhejiang University, Hangzhou, 310006 Zhejiang China; 2 Department of Prosthodontics, The Second Affiliated Hospital of Zhejiang University School of Medicine, Zhejiang, Hangzhou, 310006 Zhejiang China; 3 Department of Stomatology, The Second Affiliated Hospital of Jiaxing University, Jiaxing, 314000 Zhejiang China

**Keywords:** Strip free gingival grafts, Collagen matrix, Free gingival grafts, Dental implants, Keratinized mucosa

## Abstract

**Background:**

The aim of this study was to assess the outcomes of the combination technique of strip free gingival grafts (SFGG) and xenogeneic collagen matrix (XCM) in augmenting the width of keratinized mucosa (KMW) around dental implants, and compare its efficacy with the historical control group (FGG).

**Methods:**

Thirteen patients with at least one site with KMW ≤ 2 mm after implant surgery were included and received SFGG in combination with XCM. Another thirteen patients with the same inclusion and exclusion criteria from the previous trial received FGG alone. The same outcomes as the previous trial were evaluated. KMW, thickness of keratinized mucosa (KMT), gingival index (GI) and probing depth (PD) were measured at baseline, 2 and 6 months. Postoperative pain, patient satisfaction and aesthetic outcomes were also assessed.

**Results:**

At 6 months after surgery, the combination technique could attain 3.3 ± 1.6 mm of KMW. No significant change could be detected in GI or PD at 6 months compared to those at 2 months (*p* > 0.05). The postoperative pain and patient satisfaction in VAS were 2.6 ± 1.2 and 9.5 ± 1.2. The total score of aesthetic outcomes was 3.8 ± 1.2. In the historical FGG group, 4.6 ± 1.6 mm of KMW was reported at 6 months, and the total score of aesthetic outcomes was higher than the combination technique (4.8 ± 0.7 vs. 3.8 ± 1.2, *p* < 0.05).

**Conclusions:**

The combination technique of SFGG and XCM could increase KMW and maintain peri-implant health. However, this combination technique was associated with inferior augmentation and aesthetic outcomes compared with FGG alone.

**Trial registration:**

This clinical trial was registered in the Chinese Clinical Trial Registry with registration number ChiCTR2200057670 on 15/03/2022.

**Supplementary Information:**

The online version contains supplementary material available at 10.1186/s12903-024-04184-y.

## Background

Nowadays, more research has focused on keratinized mucosa around dental implants, which has been considered to play a vital role in maintaining peri-implant health [1]. There is plenty of evidence that inadequate keratinized mucosa width (KMW) (≤ 2 mm) is associated with more plaque accumulation, soft tissue inflammation, mucosal recession, and marginal bone loss [2–8].

To obtain adequate KMW, various options exist: the use of an apically repositioned flap (ARF) alone, the use of a combination of ARF and autologous tissue grafts or other substitutes, or the strip gingival graft technique. To date, ARF in combination with autologous tissue grafts appears to be the most predictable and stable approach for augmenting KMW around dental implants [9–11]. Our previous study has demonstrated that ARF plus free gingival grafts (FGG) could result in a greater increase of KMW than ARF plus xenogeneic collagen matrix (XCM), though both could attain > 2 mm KMW [12].

However, harvesting of a soft tissue graft may indicate more postoperative pain and discomfort [13]. In addition, the size of autologous soft tissue grafts harvested is relatively limited due to the existence of the greater palatine neurovascular bundle [14, 15]. On the other hand, significant shrinkage of the augmented keratinized tissue after grafting with XCM alone has been reported in the literature, varying from 35.82-75% [16–18]. Thereby, Urban et al. has introduced a new technique which combines a strip free gingival graft (SFGG) with XCM to decrease the need for harvesting an extensive autologous graft and prevent the rebound of the apically repositioned mucosa [19]. More specifically, this technique utilizes SFGG fixed to the most apical location of the periosteal bed as a mechanical barrier and cell source. XCM, a porcine-derived non-cross-linked absorbable bio-membrane [20], is used to cover the remaining periosteal bed. A human histologic study evaluated the biopsy samples from the soft tissue treated with a combination of SFGG and XCM [21]. At 12 months, tissue morphology, expression levels of keratin and collagen appeared similar with reference samples of palatal autogenous grafts, which confirmed that the combination of SFGG and XCM provides physiologically normal keratinized mucosa [21].

But to the best of the author’s knowledge, clinical data on the combination technique of SFGG and XCM are very limited. Only a case series study reported favorable outcomes that a mean KMW of 6.33 mm was attained at sites treated with SFGG plus XCM at 12 months [19]. Based on this fact, the outcomes of the combination technique need to be further investigated. A single-arm trial is a trial in which all patients receive the same intervention and usually use historical clinical trial data as an external control arm. Our previous study was a randomized, controlled, parallel-group clinical trial, in which included patients were divided into the test group (XCM) and control group (FGG) [12]. Therefore, this single-arm clinical trial aimed to assess the clinical outcomes and patient-centered outcomes of the combination technique of SFGG and XCM in augmenting KMW around dental implants, and meanwhile compare the efficacy of this combination technique with the historical control group (FGG), in which the same inclusion and exclusion criteria as the previous study were applied and the same outcomes were evaluated.

## Methods

This prospective, single-arm, open clinical trial was performed in the Affiliated Stomatology Hospital of Zhejiang University School of Medicine and registered in the Chinese Clinical Trial Registry with registration number ChiCTR2200057670 on 15/03/2022. The study was conducted in accordance with the Declaration of Helsinki as revised in 2013 and approved by Stomatology Research Ethics Committee of the Affiliated Stomatology Hospital of Zhejiang University School of Medicine (No.2022-023). All participants provided written informed consent.

### Participants

Patients who met the following inclusion criteria were included by one investigator (J. P. H.): no less than 18 years old, presence of at least one site with KMW ≤ 2 mm after implant surgery and before prosthetic treatment, and need for keratinized mucosa augmentation for aesthetic or functional reasons. The exclusion criteria were smokers, pregnant or lactating women, presence of active periodontal diseases, presence of systemic disorders or medication usage interfering with mucosal healing, undergoing radiotherapy, allergy to collagen, and history of mucogingival surgery.

### Study design

In this single-arm clinical trial, all participants received SFGG plus XCM. The control group received FGG alone, which was a historical control borrowed from our previous randomized controlled clinical trial [12]. In that trial, participants were included with the same inclusion and exclusion criteria, and randomized into FGG group or XCM group.

All surgical procedures were carried out under local anesthesia by the same experienced surgeon (P.H.D.) and described as follows: first, a split-thickness flap was elevated around 10 mm apically with the help of a horizontal incision slightly coronal to the mucogingival junction (MGJ) and two vertical releasing incisions using a #15 C blade. Next, the flap was displaced apically beyond the MGJ and fixed to the periosteum using 5 − 0 resorbable T-mattress sutures (Ethicon J&J, New Brunswick, NJ). A periodontal probe (UNC 15; Hu-Friedy, Chicago, IL) was used to measure the size of the recipient bed. In the present test group, a 1–1.5 mm thick and 3 mm wide SFGG harvested from the palate was sutured to the apical region of the recipient site with 5 − 0 non-resorbable nanofilament, single-interrupted sutures and cross-mattress sutures (Ethicon J&J). Then, the collagen matrix (Mucograft®; Geistlich Pharma AG, Wolhusen, Switzerland) was trimmed into proper size to cover the remaining recipient bed (Figure S1). For the historical control group, only a 1–1.5 mm thick and suitable wide FGG was fixed to the recipient bed using 5–0 non-resorbable nanofilament single-interrupted sutures and sling sutures.

Postoperative medication included antibiotics (250 mg cefuroxime axetil, b.i.d., 6 days), analgesics (250 mg paracetamol in case of pain) and mouthwash (10mL 0.12% chlorhexidine, t.i.d., 2 weeks). Participants were also advised to adhere to a soft-food diet and brush using a soft-bristled toothbrush except the surgical site. Sutures of the palatal donor site and recipient site were removed after 1 week and 2 weeks, respectively. Follow-up examinations were performed at 2 and 6 months.

### Outcome assessments

The primary outcome and secondary outcomes in the present study were the same as the previous clinical trial [12].

KMW was the primary outcome of this study, which was measured from the MGJ to the mucosa margin or the zenith of ridge at the mid-buccal aspect using a periodontal probe (UNC15) at baseline, 2 months and 6 months after surgery.

Keratinized mucosa thickness (KMT), gingival index (GI) and probing depth (PD) were also measured at baseline, 2 months and 6 months as previously described [12]. KMT was evaluated at the mid-point of an apical-coronal direction using an endodontic file with a rubber stop. GI was assessed at 4 sites (mesio-buccal, mid-buccal, disto-buccal, and lingual) according to the index of Loe and Silness. PD was measured using a UNC15 periodontal probe at 6 sites, including mesio-buccal, mid-buccal, disto-buccal, mesio-lingual, mid-lingual, and disto-lingual. The time between the first incision and the last suture was recorded as operation time. To evaluate post-operative pain, a 100-mm visual analog scale (VAS) ranging from 0 to 10 was applied at 1 week. VAS scores (0–10) for patient satisfaction were recorded at 2 and 6 months. At 6 months, aesthetic outcomes were rated on the color, contour, and texture of the surgical sites to give a total score, which varied between 0 and 6 by scoring each subject from 0 to 2.

### Sample size

The sample size of each group was set at 13, referring to the previous randomized controlled clinical trial [12]. Briefly, α = 0.05, power = 90% and 𝜎 =$\sqrt{\frac{{{\sigma }_{1}}^{2}+{{\sigma }_{2}}^{2}}{2}}$ = 0.77 mm (𝜎1 = 0.64 mm, 𝜎2 = 0.88 mm) [22] were used. The minimum clinically significant difference in KMW was assumed as 1.0 mm. Based on the above data, 11 patients for each group was needed to be included. Further, 13 patients for each group was determined to compensate for 10% of possible drop-outs.

### Statistical analysis

SPSS 24.0 software (SPSS, Chicago, IL) was used for statistical analysis. Categorical variables were presented as frequency (percentage) and two groups were compared using the χ2 test or Fisher’s exact test when appropriate. In terms of continuous variables, data were presented as mean (standard deviation). The Shapiro–Wilk test was applied for examination of a normal distribution. The difference between 2-month and 6-month follow-up in the same group was analyzed by the paired t-test if normally distributed or Wilcoxon signed rank test otherwise. For inter-group comparison, KMW, KMT, GI, PD and aesthetic outcomes were analyzed using multilevel models. To compare operation time and patient-centered outcomes between two groups, the independent t-test was performed if normally distributed and the Mann–Whitney test was used if otherwise. A two-tailed *p*-value less than 0.05 was statistically significant.

## Results

**Fig. 1:**
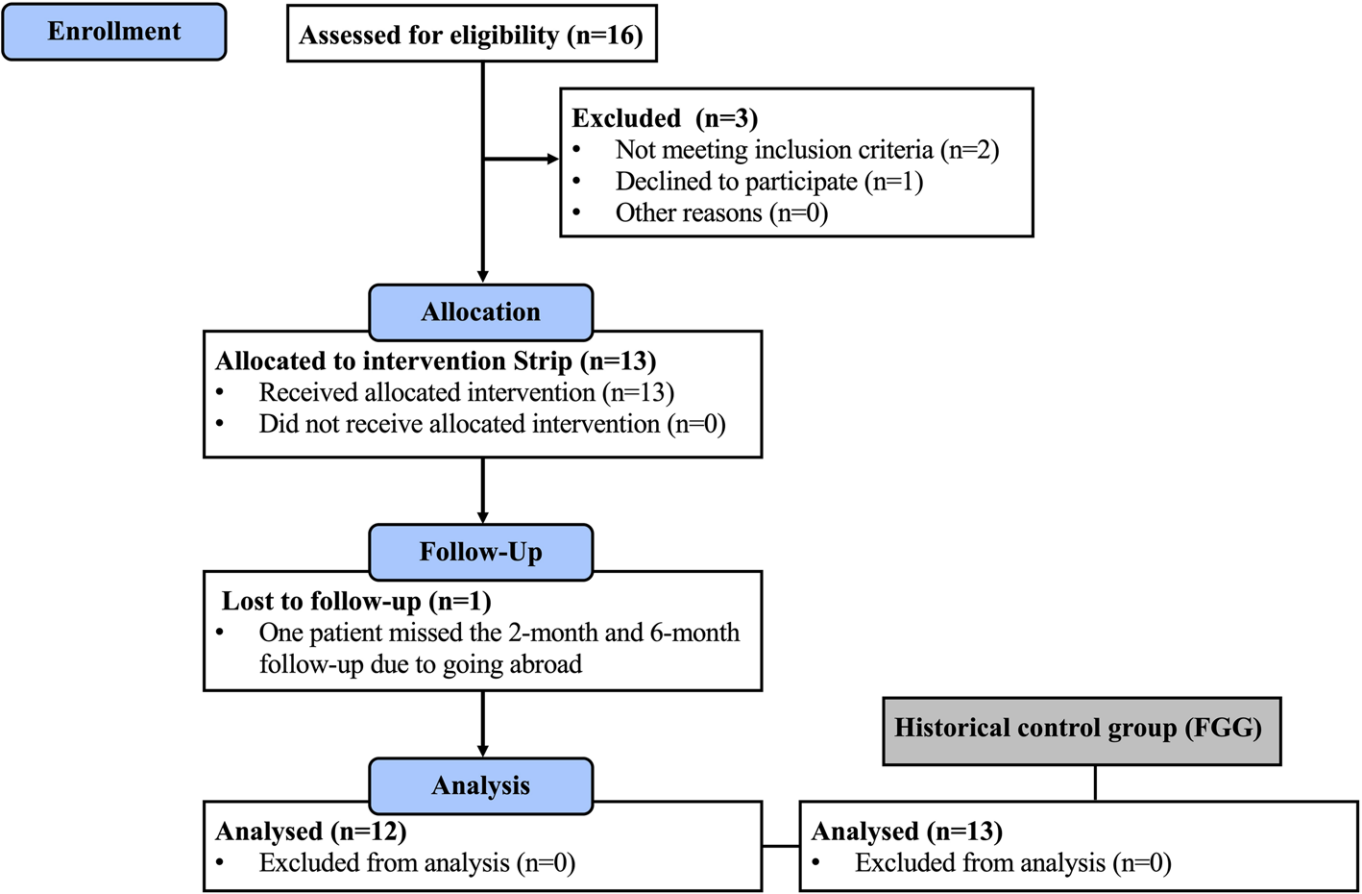
The flowchart of patient enrollment, allocation, follow-up and analysis

As shown in Fig. 1, thirteen participants (five males and eight females) were included in the Strip group. Information including demographic data and clinical characteristics at baseline were presented in Table 1. The mean age was 59 ± 7 years. The majority of surgical sites were located in the posterior area (73%) and mandible (60%). In the Strip group, KMW and KMT at baseline were 0.5 ± 0.6 and 0.9 ± 0.2 mm. Comparing Strip group with the historical control group (FGG), no statistically significant difference was observed in gender, brand distribution of implants, KMW or KMT at baseline. All included patients received SFGG in combination with XCM. However, one patient missed the 2-month and 6-month follow-up due to going abroad. Recruitment, interventions and follow-up visits were performed from March 2022 to May 2023.

**Table 1:** Demographic data and clinical characteristics at baseline [12]

**Variable**	**Group FGG**	**Group Strip**	***p-*Value**
Patients			
N	13	13	
Age (years)	49 (13)	59 (7)	0.039*
Gender			
Male	5 (38%)	5 (38%)	1.000
Female	8 (62%)	8 (62%)	
Implants			
n	19	15	
Area			
Anterior	1 (5%)	4 (27%)	0.146
Posterior	18 (95%)	11 (73%)	
Jaw			
Maxilla	0 (0%)	6 (40%)	0.004**
Mandible	19 (100%)	9 (60%)	
Brands			
Straumann	14 (74%)	15 (100%)	0.113
Astra	4 (21%)	0 (0%)	
Camlog	1 (5%)	0 (0%)	
Clinical characteristics			
KMW (mm)	0.5 (0.6)	0.3 (0.4)	0.221
KMT (mm)	0.9 (0.2)	1.1 (0.5)	0.595

*Abbreviations* *FGG* free gingival graft, *KMW* width of keratinized mucosa, *KMT* thickness of keratinized mucosa, *N* number of patients, *n* number of implants. Data were presented as mean (SD) or number (%)

*indicates *p* < 0.05

**indicates *p* < 0.01

KMW was the primary outcome of this study, which was presented in Table 2. The mean KMW of the Strip group at 2 and 6 months after surgery were 3.7 ± 2.2 mm and 3.3 ± 1.6 mm. They seemed to be numerically lower than those of the FGG group (4.6 ± 1.5 mm and 4.6 ± 1.6 mm), but without significant difference (both *p* > 0.05). Meanwhile, there was no significant difference in the change of KMW between two groups at 2-month or 6-month follow-up (*p* > 0.05). It was noteworthy that KMW evaluated at 6 months was lesser than that at 2 months in the Strip group (3.3 ± 1.6 mm vs. 3.7 ± 2.2 mm, *p* < 0.05).

**Table 2:** Comparison of KMW between Group FGG and Group Strip at 2 and 6 months [12]

	**Group FGG**			**Group Strip**			**Inter-group** ***p*-Value**
	**N**	**n**	**KMW (mm)**	**N**	**n**	**KMW (mm)**	
2 months	13	19	4.6 (1.5)	12	14	3.7 (2.2)	0.337
6 months	13	19	4.6 (1.6)	12	14	3.3 (1.6)	0.100
2 months-Baseline	13	19	4.1 (1.4)	12	14	3.4 (2.2)	0.471
6 months-Baseline	13	19	4.1 (1.6)	12	14	3.0 (1.6)	0.173
Intra-group *p*-Value			1.000			0.026*	

*Abbreviations*: *FGG* free gingival graft, *KMW* width of keratinized mucosa, *N* number of patients, *n* number of implants. Data were presented as mean (SD)

*indicates *p*< 0.05

As shown in Table 3, the mean KMT at 2-month and 6-month follow-up in the Strip group were both 1.7 ± 0.4 mm. No significant difference could be observed in KMT or the change of KMT between two groups (*p* > 0.05). In addition, no significant change was found in GI or PD at 6 months compared to those at 2 months in the Strip group (*p* > 0.05) (Table 4).

**Table 3:** Comparison of KMT between Group FGG and Group Strip at 2 and 6 months [12]

	**Group FGG**			**Group Strip**			**Inter-group** ***p*-Value**
	**N**	**n**	**KMT (mm)**	**N**	**n**	**KMT (mm)**	
2 months	13	19	1.8 (0.5)	12	14	1.7 (0.4)	0.378
6 months	13	19	1.7 (0.6)	12	14	1.7 (0.4)	0.986
2 months-Baseline	7	10	1.0 (0.3)	5	5	0.8 (0.7)	0.580
6 months-Baseline	7	10	0.9 (0.5)	5	5	0.8 (0.7)	0.940
Intra-group *p*-Value			0.457			0.197	

*Abbreviations*: *FGG* free gingival graft, *KMT* thickness of keratinized mucosa, *N* number of patients, *n* number of implants. Data were presented as mean (SD).

**Table 4:** Comparison of GI and PD between Group FGG and Group Strip at 2 and 6 months [12]

			**Group FGG**				**Group Strip**		**Inter-group *p*-Value**	
	**N**	**n**	**GI**	**PD (mm)**	**N**	**n**	**GI**	**PD (mm)**	**GI**	**PD **
2 months	13	19	0.14 (0.28)	1.41 (0.39)	12	14	0.14 (0.13)	1.61 (0.50)	0.982	0.217
6 months	13	19	0.11 (0.27)	1.36 (0.35)	12	14	0.18 (0.15)	1.53 (0.44)	0.366	0.281
Intra-group *p*-Value			0.581	0.715			0.527	0.612		

*Abbreviations*: *FGG* free gingival graft, *GI* gingival index, *PD* probing depth, *N* number of patients, *n* number of implants. Data were presented as mean (SD)

In terms of aesthetic outcomes, the contour score of the Strip group (0.8 ± 0.5) was lower than its color score (1.5 ± 0.5) and texture score (1.5 ± 0.6) (Table 5). Compared with the FGG group, the contour score and texture score of the Strip group was significantly lower (0.8 ± 0.5 vs. 1.4 ± 0.4, *p* < 0.01; 1.5 ± 0.6 vs. 1.9 ± 0.3, *p* < 0.05), which might be accounted for the lower total score of the Strip group (3.8 ± 1.2 vs. 4.8 ± 0.7, *p* < 0.05). Regarding the operation time, there was no significant difference between the Strip group and FGG group (60 ± 4 min vs. 60 ± 9 min, *p* > 0.05) (Table 5). In the Strip group, the post-operative pain and dose of paracetamol were 2.6 ± 1.2 and 385 ± 282 mg, respectively (Table 5). The post-operative pain and dose of paracetamol in the FGG group were 3.4 ± 1.8 and 942 ± 785 mg (Table 5).

**Table 5:** Comparison of aesthetic outcomes and patient-centered outcomes between Group FGG and Group Strip [12]

**Variable**	**Group FGG**			**Group Strip**			**Inter-group *p*-Value**
	**N**	**n**		**N**	**n**		
Aesthetic outcomes	13	19		12	14		
color			1.4 (0.6)			1.5 (0.5)	0.513
contour			1.4 (0.4)			0.8 (0.5)	0.003**
Texture			1.9 (0.3)			1.5 (0.6)	0.014*
total			4.8 (0.7)			3.8 (1.2)	0.024*
Operation time	13		60 (9)	13		60 (4)	0.920
Postoperative pain in VAS	13		3.4 (1.8)	13		2.6 (1.2)	0.204
Dose of compound paracetamol (mg)	13		942 (785)	13		385 (282)	0.091
Patient satisfaction in VAS							
2 months	13		9.1 (0.9)	12		9.5 (1.2)	0.123
6 months	13		9.6 (0.6)	12		9.5 (1.2)	0.503

*Abbreviations*: *FGG* free gingival graft, *N* number of patients, *n* number of implants, *VAS* visual analogue scale. Data were presented as mean (SD)

*indicates *p* < 0.05

**indicates *p* < 0.01

## Discussion

The current study is a single-arm clinical trial, in which all participants received the same new intervention–SFGG in combination with XCM. This study investigated the efficacy of the combination technique of SFGG and XCM in augmenting keratinized mucosa around dental implants. Besides, this study compared the outcomes of the combination technique with the historical FGG group, in which the same inclusion criteria, exclusion criteria, outcome measurements as the previous study were applied.

The results of this study demonstrated that the combination technique could increase an average KMW of 3.0 mm at 6 months. However, Urban et al. [19] found that the combination technique of SFGG and XCM attained a mean width of 6.45 mm after 6 months, which was nearly twice as large as the average increase of KMW in our study. A plausible explanation for this discrepancy was that the majority of surgical sites in the previous study by Urban were located in the maxilla, while most of surgical interventions in our study were carried out in the posterior mandible. In the edentulous posterior mandible, there usually exist displacement of tissue attachments to the alveolar crest and reduction in vestibular depth [23]. For this reason, the efficacy of this combination technique especially XCM in regenerating keratinized mucosa may be diminished [24]. In addition, it was noteworthy that there was a slight decrease of KMW between 2 and 6 months in the Strip group. Likewise, Urban et al. [19] reported a mean gain of 6.88 mm in KMW at 3 months and 6.45 mm at 6 months following keratinized mucosa augmentation by the combination technique, which coincided with our finding.

When comparing with the historical FGG group from the previous study, no statistically significant difference was detected between FGG and SFGG plus XCM in the KMW attained at 6 months after surgery (3.3 mm vs. 4.6 mm). Nonetheless, the difference of more than 1 mm in value may still have some effect on the surgical decision-making. Our previous study revealed the inferior performance of XCM than FGG in increasing KMW around dental implants [12]. It is well known that XCM is a cell-free three-dimensional scaffold, whose regenerative outcomes in augmenting keratinized mucosa depends on keratinocytes and fibroblasts from the adjacent tissue [25]. The cell-free property and larger shrinkage rate of XCM may lead to the inferior efficacy of the combination technique compared to FGG alone [18].

With regard to KMT, the results of this study showed that a mean KMT of 1.7 mm was attained in the Strip group at 6 months. However, it must be explained here that KMT in this study was measured at the mid-point of an apical-coronal direction using an endodontic file with a rubber stop, referring to our previous study [12]. A noticeable distinction in thickness could be found between the keratinized mucosa regenerated by apically fixed SFGG and the keratinized mucosa regenerated by XCM in our study. The corresponding point for the measurement of KMT might be located in the regenerated mucosa derived from SFGG or XCM. With this respect in mind, the previous measurement may not be applicable to the Strip group. Therefore, a novel method for comprehensive evaluation of mucosa thickness should be established. For the Strip group, there was no significant difference in GI or PD at 2 and 6 months, suggesting that healthy peri-implant soft tissue could be maintained after surgery. However, it is unfortunate that GI or PD at baseline were not presented in the manuscript, as they could not be evaluated in submerged implants.

It is generally accepted that free gingival grafts match poorly with the adjacent tissue due to its characteristic to retain the original appearance of palate [26, 27]. Though FGG was utilized in both the Strip group and FGG group, the results of our study showed that the overall score, contour score and texture score of the Strip group were inferior at 6-month follow-up. This may be partly explained by the observation that the regenerated keratinized tissue derived from a palatal SFGG was distinctively different from both lateral tissue and coronal tissue. Recently, a new technique involving a labial strip gingival graft in combination with XCM has been proposed and demonstrated to be able to attain 6.8 mm of KMW after 12 months, which was similar with the KMW gain of the combination of a palatal SFGG and XCM [28]. Moreover, a better color match of the regenerated keratinized mucosa with the adjacent tissue was noted in sites with this technique [28], implying that labial gingival grafts from the adjacent area may be relevant to superior aesthetic outcomes. Nevertheless, harvesting of a labial gingival graft requires sufficient width of keratinized gingiva on the adjacent labial site, otherwise the risk of gingival recessions may be high in the cases without adequate KMW.

Another finding is the relatively low postoperative pain in VAS and dose of paracetamol. The combination technique avoids the harvesting of a large number of grafts, which can theoretically reduce postoperative pain and analgesic intake. It has been reported that the larger graft dimension (height > 4 mm, width ≥ 14 mm and thickness > 2 mm) may be related to higher postoperative discomfort and pain [29–32].

In the present study, the historical control group from the prior randomized clinical trial was borrowed in the statistical analysis to compare the efficacy of the combination technique with FGG alone. Although the same inclusion criteria, exclusion criteria and outcome measurements were applied in this single-arm trial, there is no doubt that the lack of randomization and blinding is an evident limitation of the historical control group. Hence, a randomized controlled clinical trial with the concurrent control is strongly suggested to conduct to avoid the potential impact of selection bias and detection bias on the treatment outcomes. On the other hand, the sample size is small and well-designed trials with more participants should be conducted in the future. Additionally, the previous method for assessing the thickness of keratinized mucosa is not suitable for the combination technique, which is also thought to be one of the limitations of this study.

## Conclusions

In conclusion, the present study revealed that the combination technique of strip free gingival grafts and xenogeneic collagen matrix could increase keratinized mucosa width around dental implants and maintain peri-implant health. However, this combination technique was associated with inferior augmentation and aesthetic outcomes compared with FGG alone.

### Supplementary Information

**Supplementary Material 1.**

## Data Availability

The datasets generated during the current study are available upon request by contact with the corresponding author.

## Abbreviations

ARF: Apically repositioned flap
FGG: Free gingival grafts
GI: Gingival index
KMT: Keratinized mucosa thickness
KMW: Keratinized mucosa width
MGJ: Mucogingival junction
PD: Probing depth
SFGG: Strip free gingival grafts
VAS: Visual analog scale
XCM: Xenogeneic collagen matrix
