# Supplementary material for: A combination technique of strip free gingival grafts and xenogeneic collagen matrix in augmenting keratinized mucosa around dental implants: a single-arm clinical trial
Source: BMC Oral Health. 2024 May 29;24:634. doi: 10.1186/s12903-024-04184-y (PMC11137898; doi:10.1186/s12903-024-04184-y)
Supplement: Supplementary file 1 — Supplementary Material 1. [file 12903_2024_4184_MOESM1_ESM.docx]

**
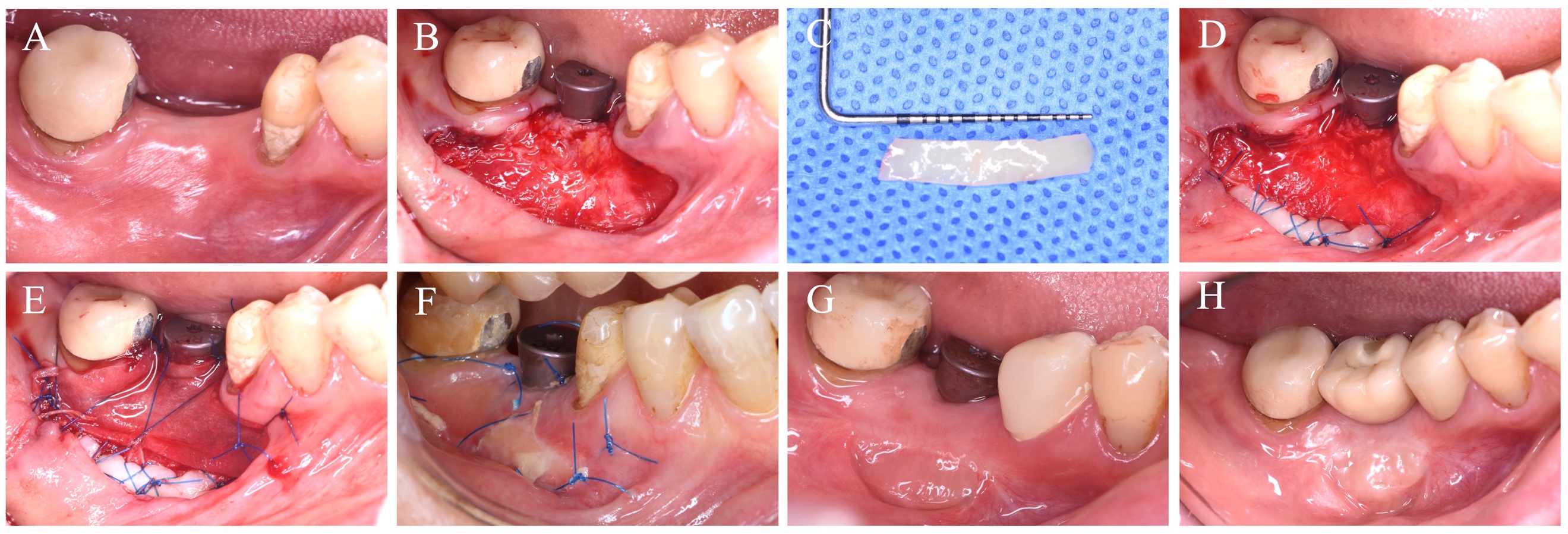
**

**Figure S1.** (A) Clinical view at baseline showing a narrow band of keratinized mucosa; (B) Split-thickness flap positioned apically and sutured to the periosteum; (C) The SFGG harvested from the palate; (D) The SFGG fixed to the apical region of the recipient bed; (E) The collagen matrix (Mucograft®) covered the remaining recipient bed; (F) Clinical view 14 days after surgery; (G) Clinical view 2 months after surgery; (H) Clinical view 6 months after surgery.
